# Supplementary material for: Titania supported synergistic palladium single atoms and nanoparticles for room temperature ketone and aldehydes hydrogenation
Source: Nat Commun. 2020 Jan 7;11:48. doi: 10.1038/s41467-019-13941-5 (PMC6946645; doi:10.1038/s41467-019-13941-5)
Supplement: Supplementary file 1 — Supplementary Information [file 41467_2019_13941_MOESM1_ESM.pdf]

Supplementary Information for

Titania Supported Synthergistic Palladium Single Atoms and Nanoparticles for Room Temperature Ketone and Aldehydes Hydrogenation

Kuai et al.

Supplementary Figure 1-25

Supplementary Table 1-3

Supplementary notes

Supplementary Reference

Supplementary Figures

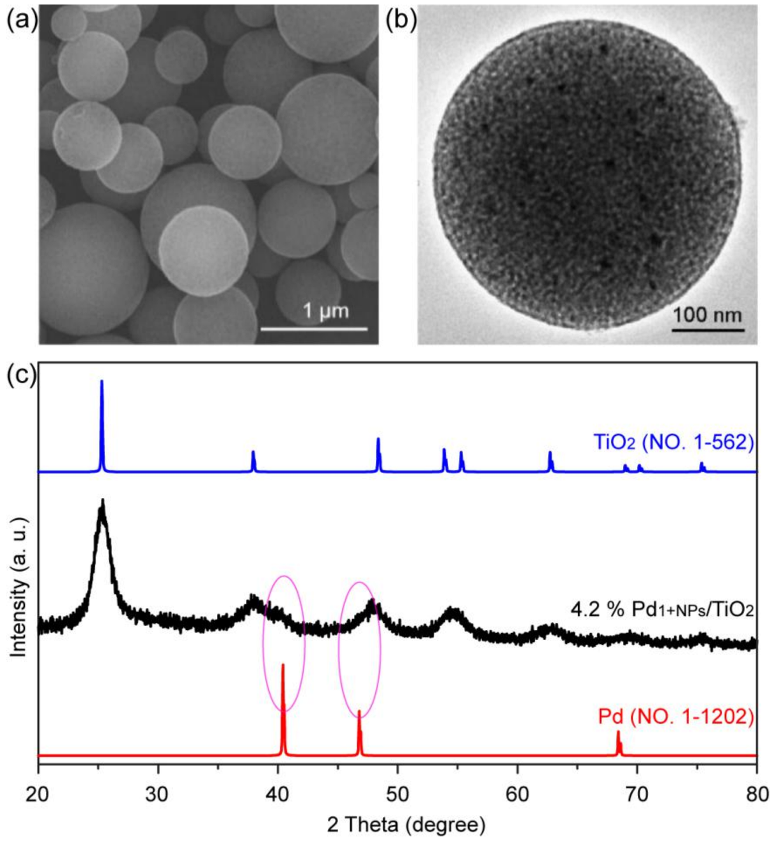

Supplementary Figure 1: The SEM (a) and TEM (b) images of Pd_{1+NPs}/TiO_2 synergistic catalyst.The XRD patterns (c) of Pd_{1+NPs}/TiO_2 (black), standard TiO_2 (blue) and Pd (red).

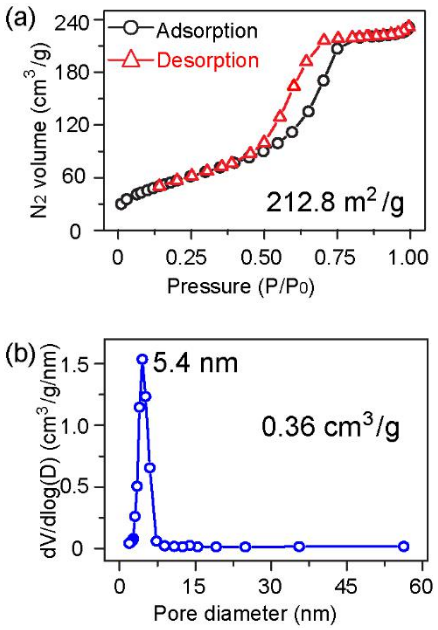

Supplementary Figure 2: N_2 adsorption/desorption isotherm (a) and BJH pore size distribution (b) of Pd_{1+NPs}/TiO_2 synergistic catalyst.

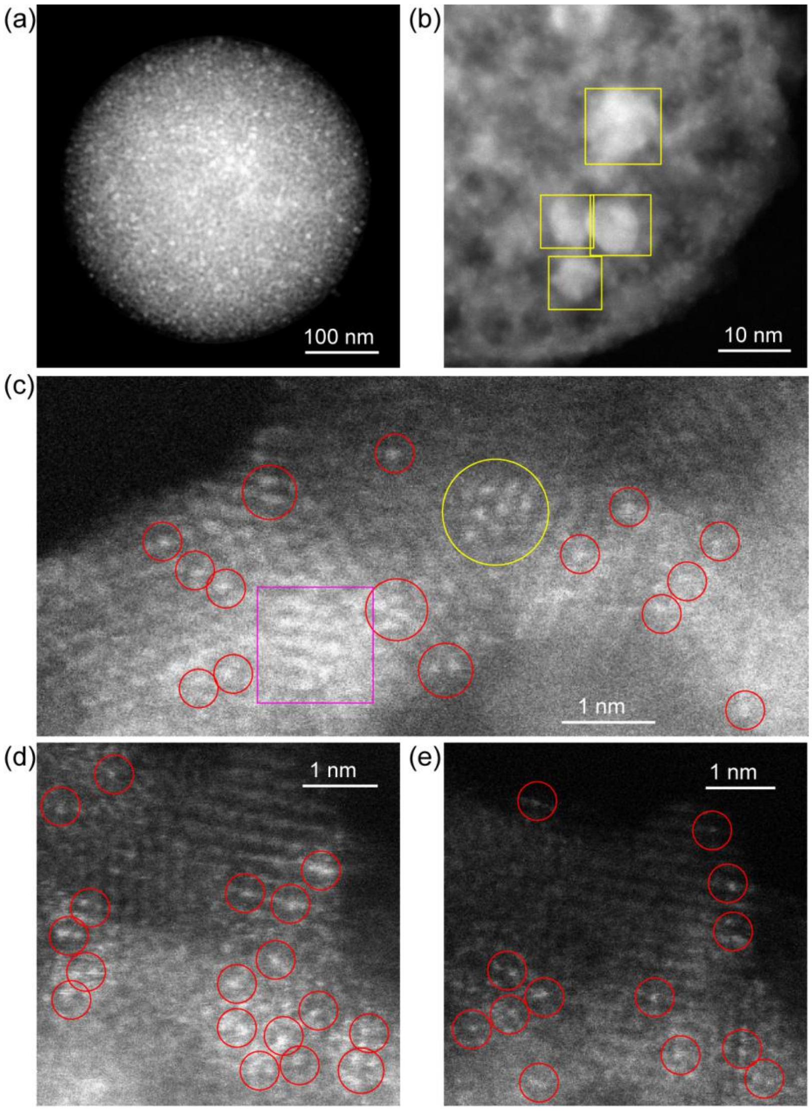

Supplementary Figure 3: Low (a) and high (b) magnification HAADF-STEM images and three typically atomically scaled images (c-e) of 4.2% Pd_{1+NPs}/TiO_2 mesoporous catalysts.

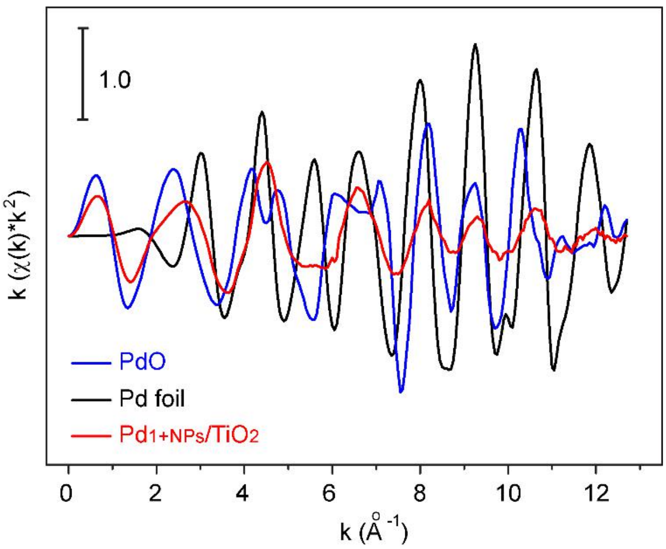

Supplementary Figure 4: K-spaced EXAFS spectra of Pd_{1+NPs}/TiO_2 synergistic catalyst (red), PdO (blue) and metallic Pd foil (black).

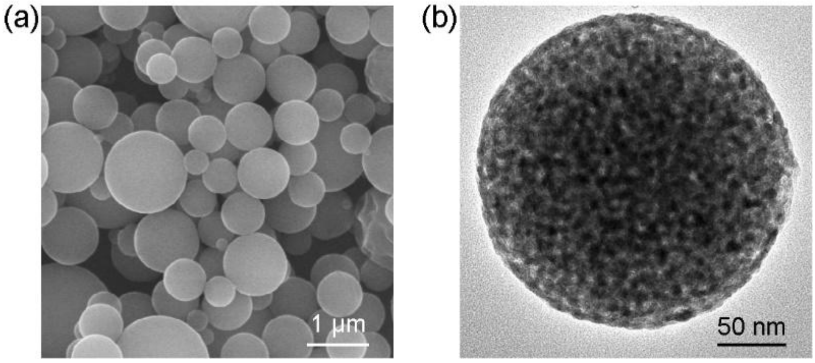

Supplementary Figure 5: The SEM (a) and TEM (b) images of pristine TiO_2 mesoporous catalysts.

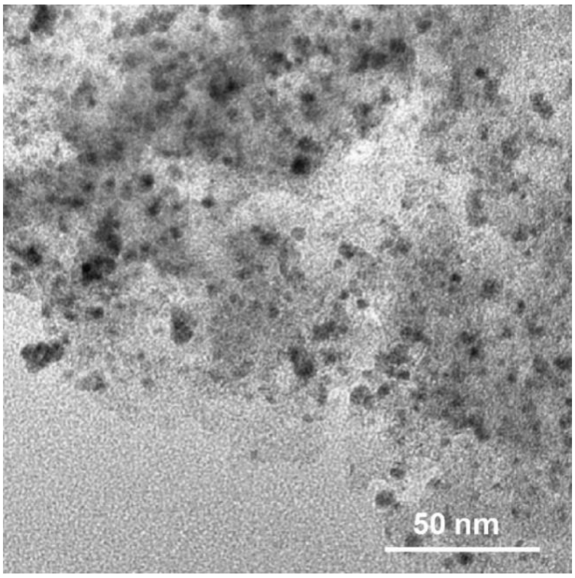

Supplementary Figure 6: The TEM image of commercial 5.2 *wt*% Pd/C catalysts purchased from Aladdin.

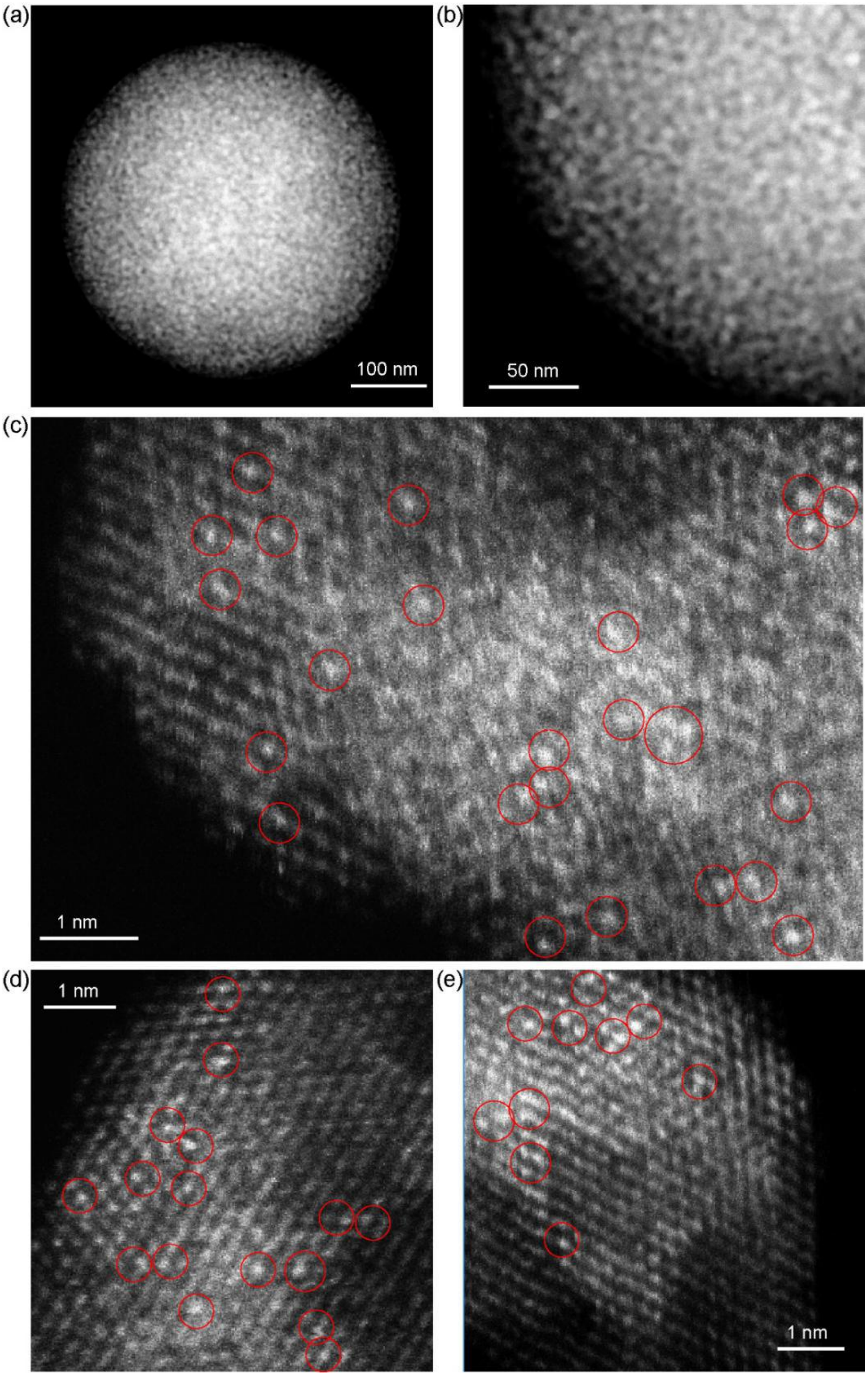

Supplementary Figure 7: Low (a) and high (b) magnification HAADF-STEM images and three typically atomically scaled images (c-e) of 1% Pd_1/TiO_2 mesoporous catalysts.

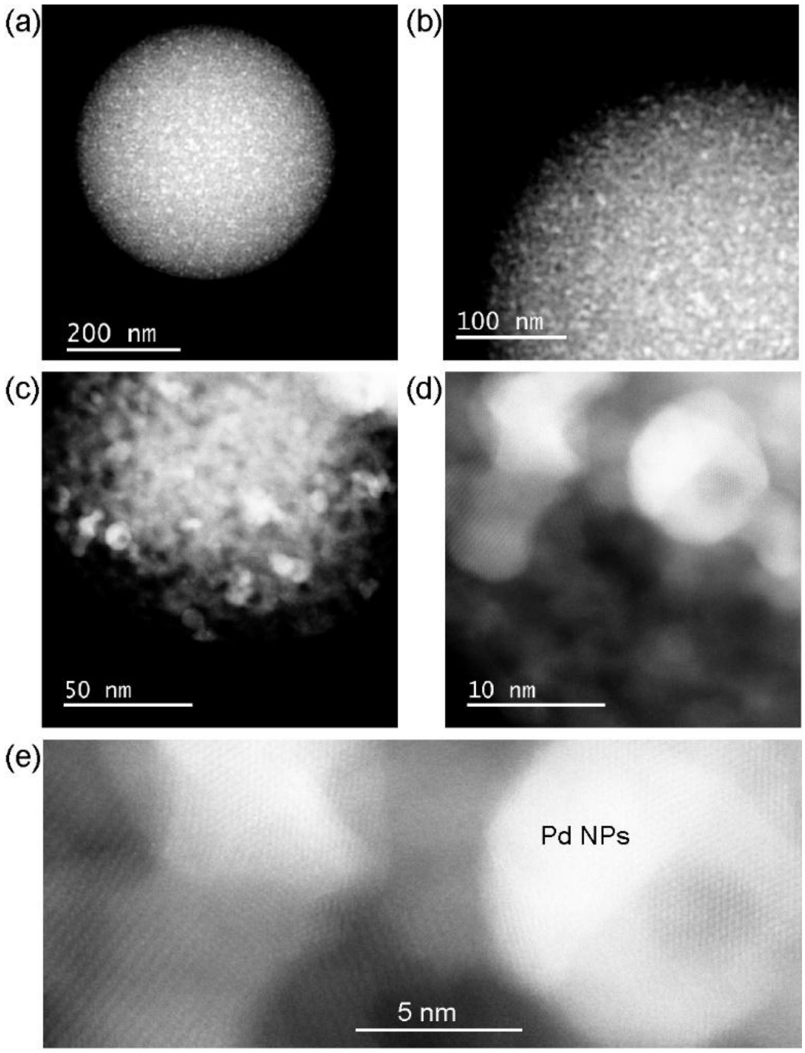

Supplementary Figure 8: The HAADF-STEM images (a-c) with increased magnification and typically atomically scaled images (d and e) of 5% Pd_{NPs}/TiO_2 mesoporous catalysts.

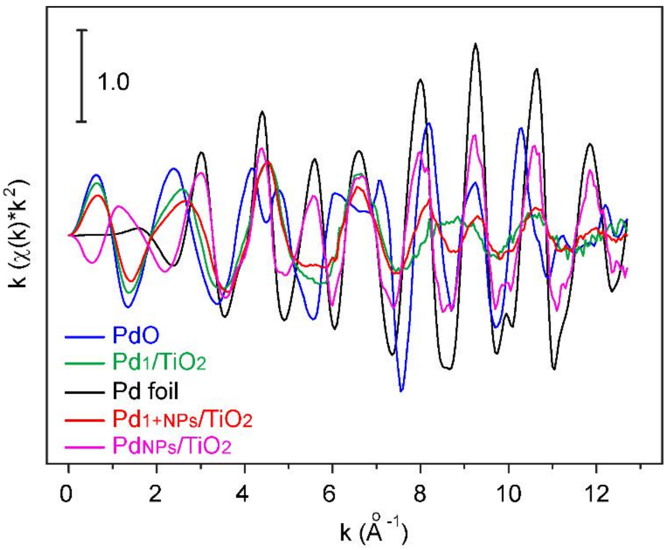

Supplementary Figure 9: K-spaced EXAFS spectra of Pd_{1+NPs}/TiO_2 synergistic catalyst (red), Pd_1/TiO_2 (green), Pd_{NPs}/TiO_2 (pink), PdO (blue) and metallic Pd foil (black).

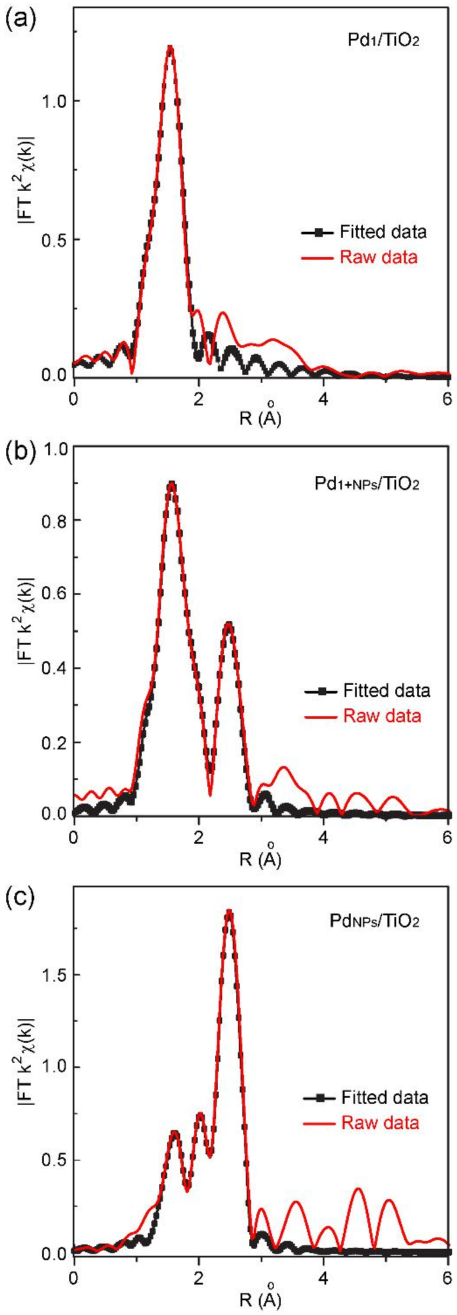

Supplementary Figure 10: The fitted R-spaced FT-EXAFS of Pd_1/TiO_2 (a), Pd_{1+NPs}/TiO_2 (b) and Pd_{NPs}/TiO_2 (c) catalysts.

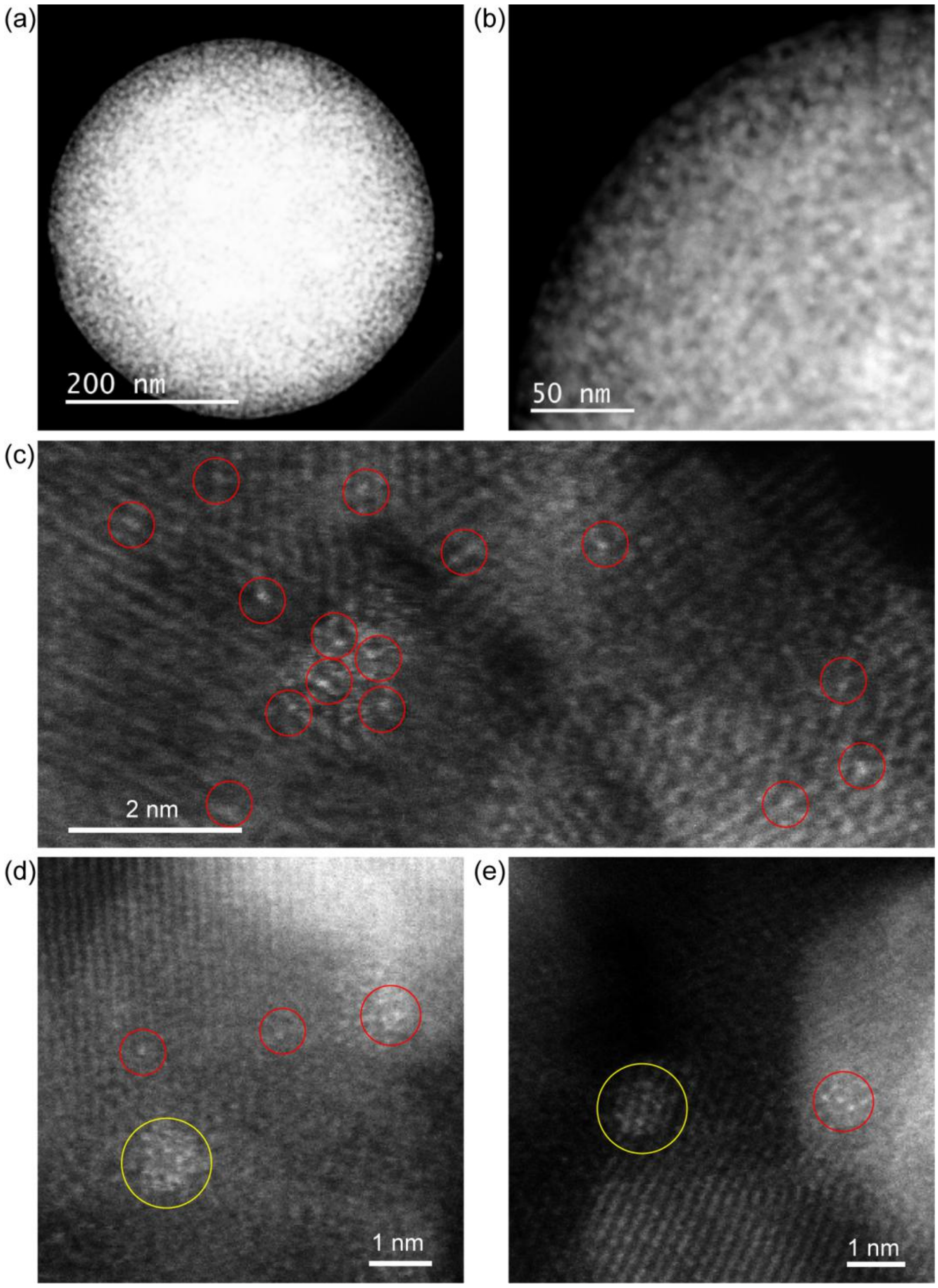

Supplementary Figure 11: Low (a) and high (b) magnification HAADF-STEM images and three typically atomically scaled images (c-e) of used 1% Pd_1/TiO_2 mesoporous catalysts with 1 hour's reaction for 4-methylacetophene hydrogenation.

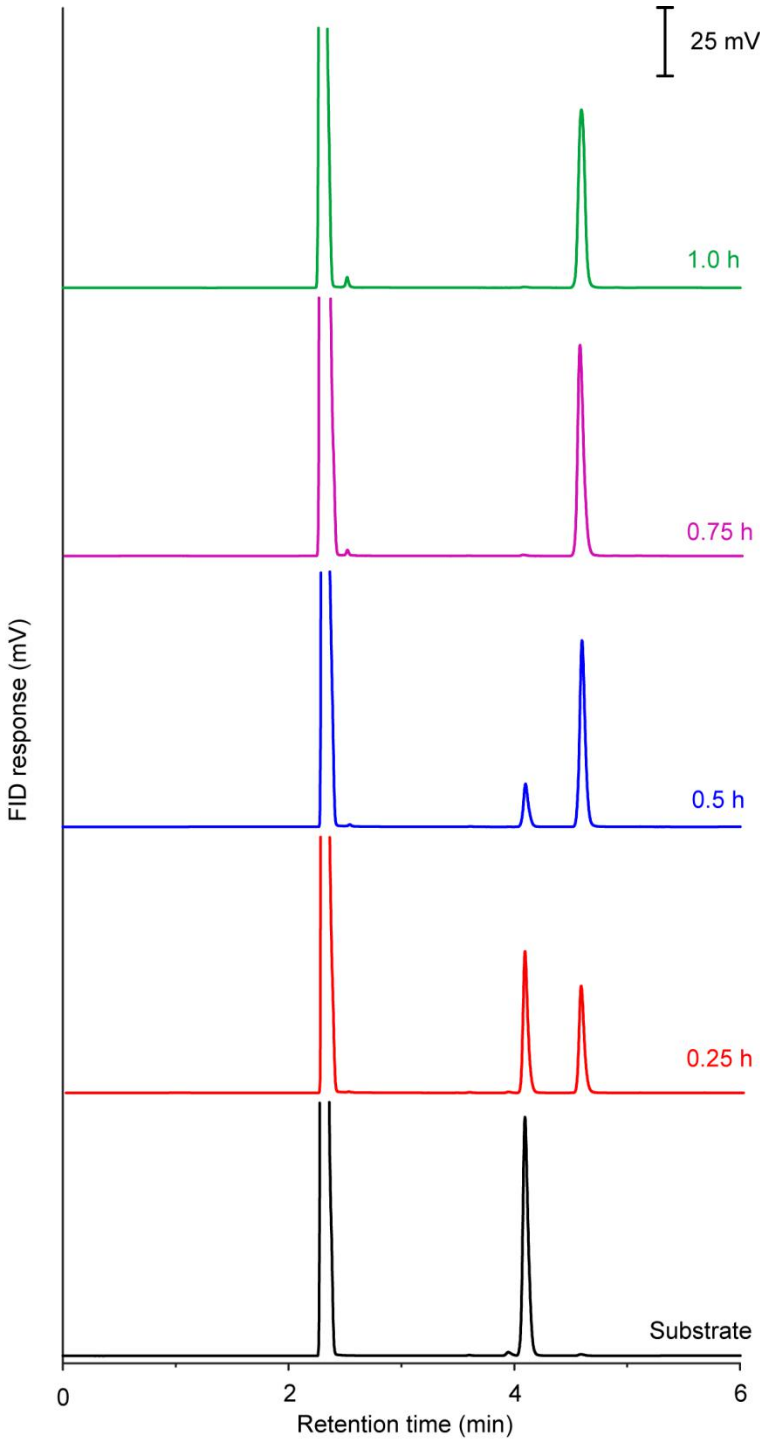

Supplementary Figure 12: The FID responded GC spectra of 4-methylacetophene substrate (black) and the products obtained at 0.25 h (red), 0.5 h (blue), 0.75 h (purple) and 1.0 h (green). GC parameters of temperature: gasification chamber (240 ^{o}C), FID detector (260 ^{o}C) and column (200 ^{o}C).

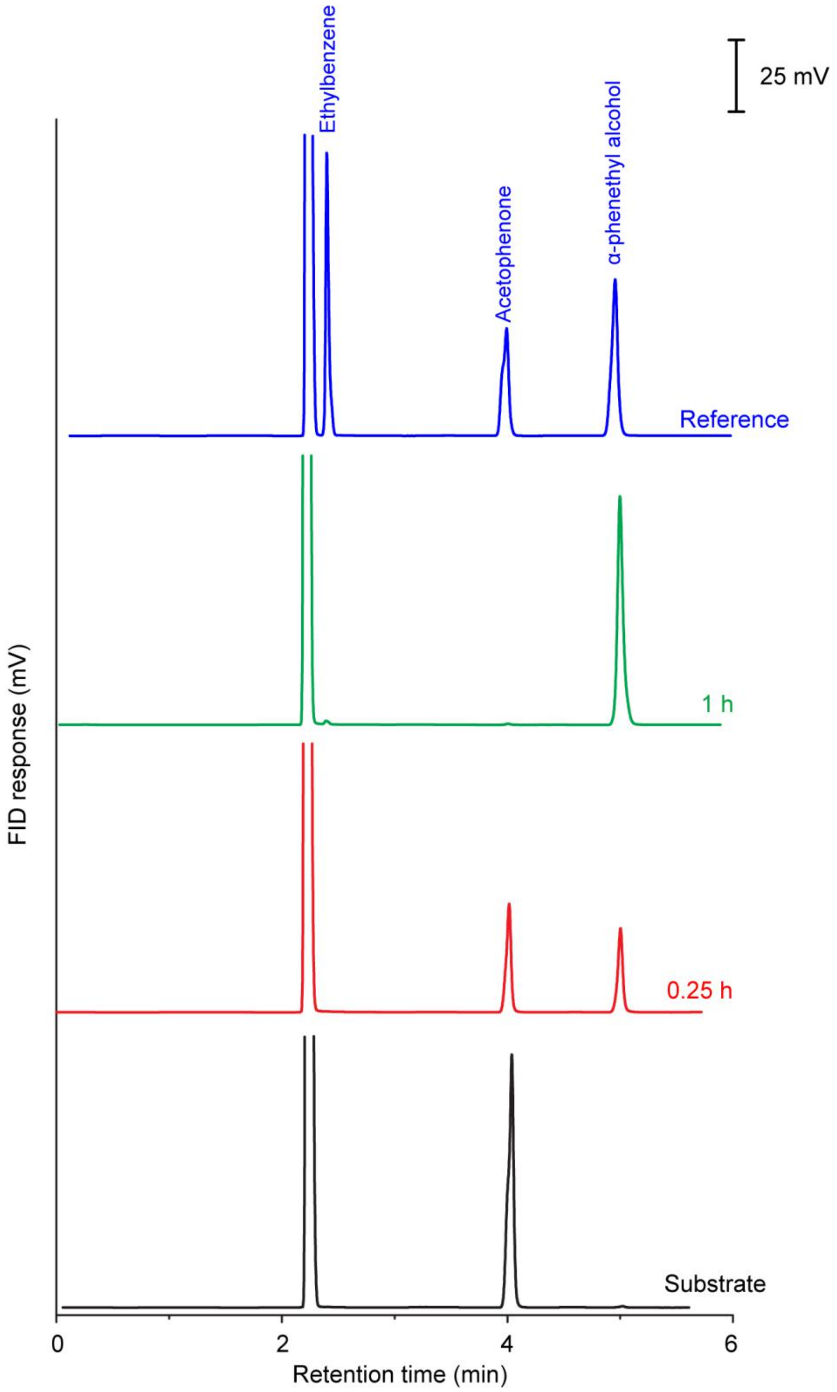

Supplementary Figure 13: The FID responded GC spectra of acetophenone substrate (black), the products obtained at 0.25 h (red) and 1.0 h (green) and reference (blue) with known substances. GC parameters of temperature: gasification chamber (240 ^{o}C), FID detector (260 ^{o}C) and column (180 ^{o}C).

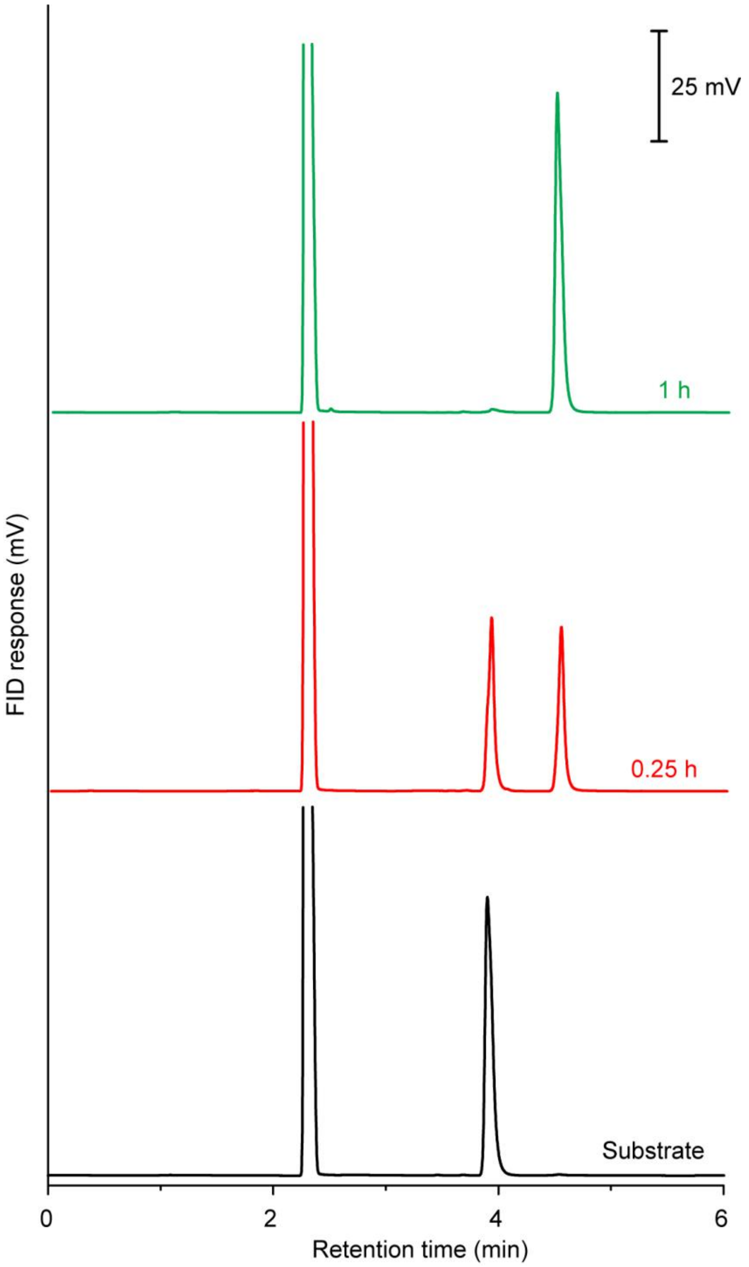

Supplementary Figure 14: The FID responded GC spectra of 3-methylacetophene substrate (black) and the products obtained at 0.25 h (red) and 1.0 h (green). GC parameters of temperature: gasification chamber (240 ^oC), FID detector (260 ^oC) and column (200 ^oC).

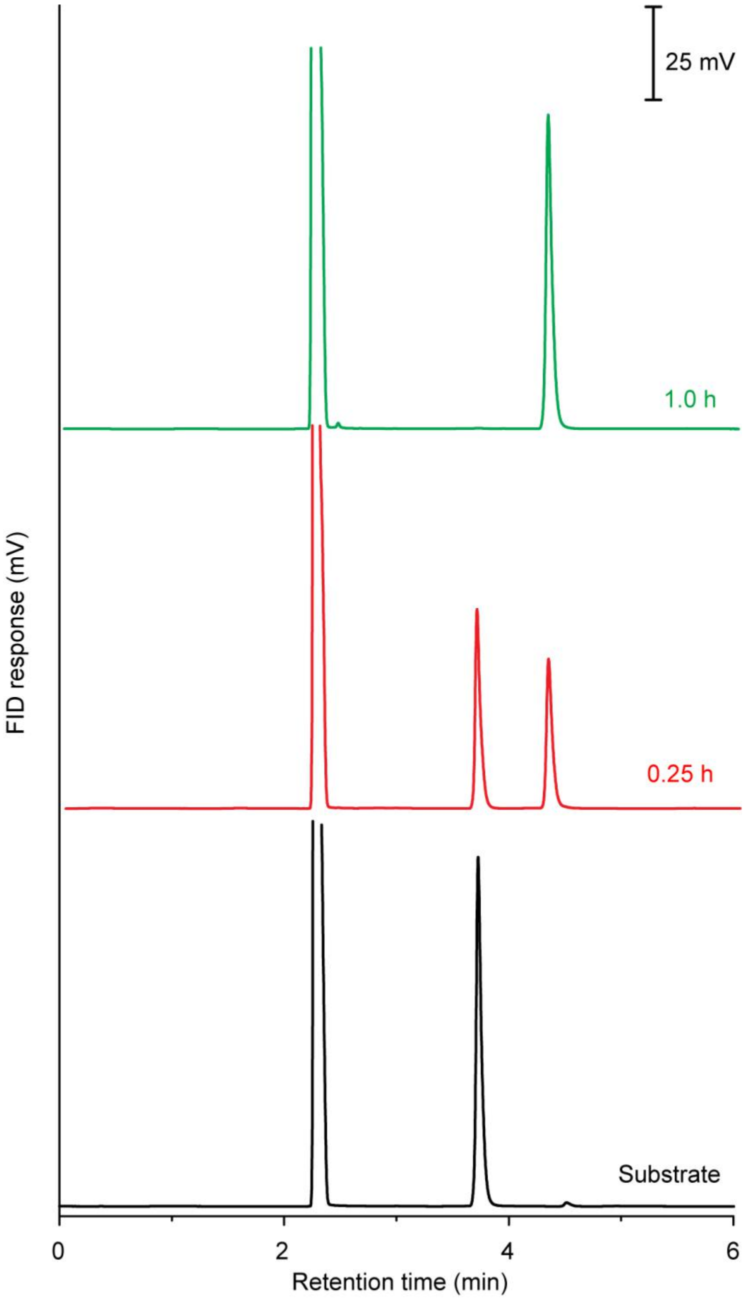

Supplementary Figure 15: The FID responded GC spectra of phenylacetone substrate (black) and the products obtained at 0.25 h (red) and 1.0 h (green). GC parameters of temperature: gasification chamber (240 ^{o}C), FID detector (260 ^{o}C) and column (200 ^{o}C).

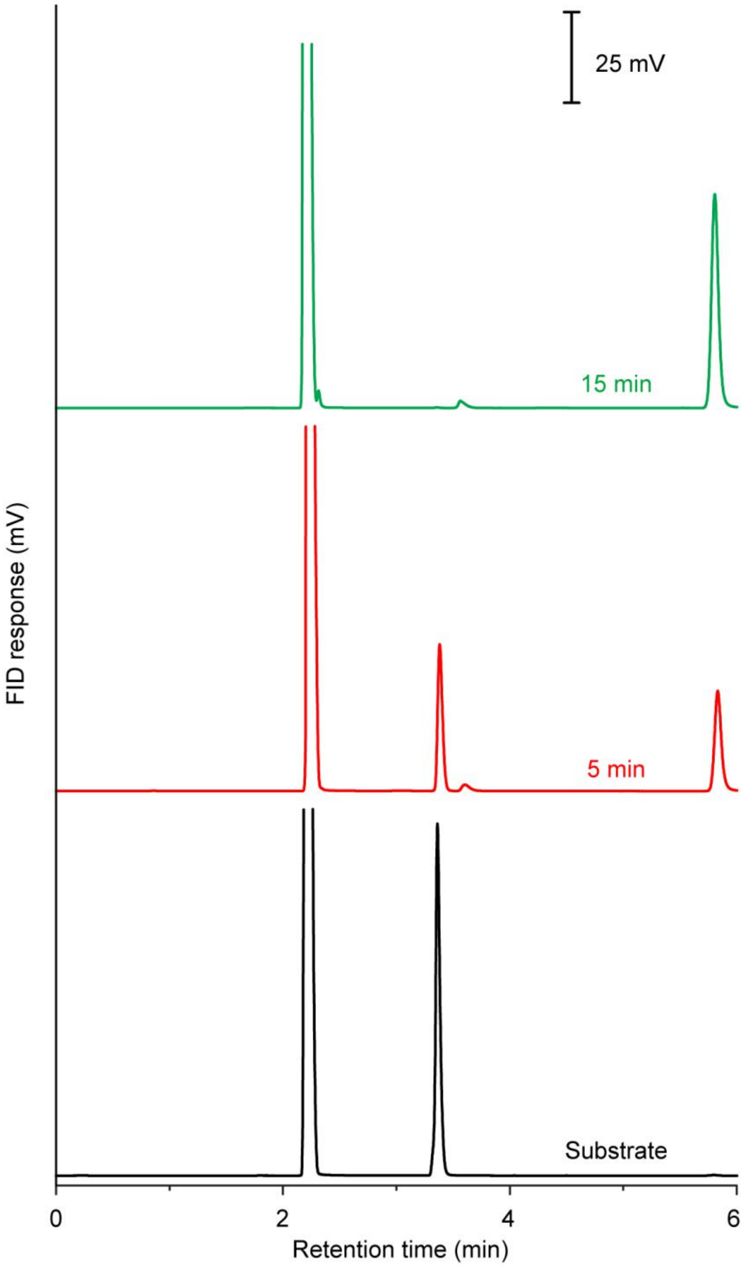

Supplementary Figure 16: The FID responded GC spectra of benzaldehyde substrate (black) and the products obtained at 5 min (red) and 15 min (green). GC parameters of temperature: gasification chamber (240 ^{o}C), FID detector (260 ^{o}C) and column (180 ^{o}C).

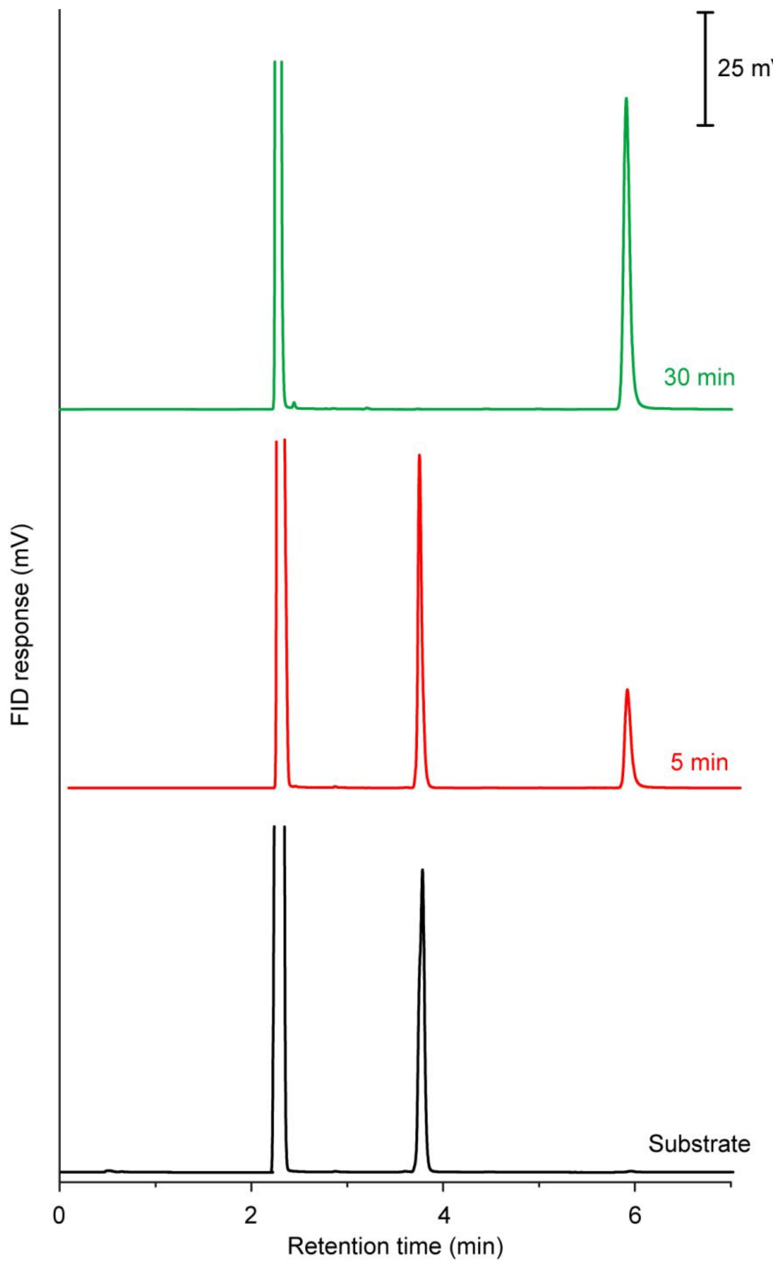

Supplementary Figure 17: The FID responded GC spectra of 4-methylbenzaldehyde substrate (black) and the products obtained at 5 min (red) and 30 min (green). GC parameters of temperature: gasification chamber (240 ºC), FID detector (260 ºC) and column (190 ºC).

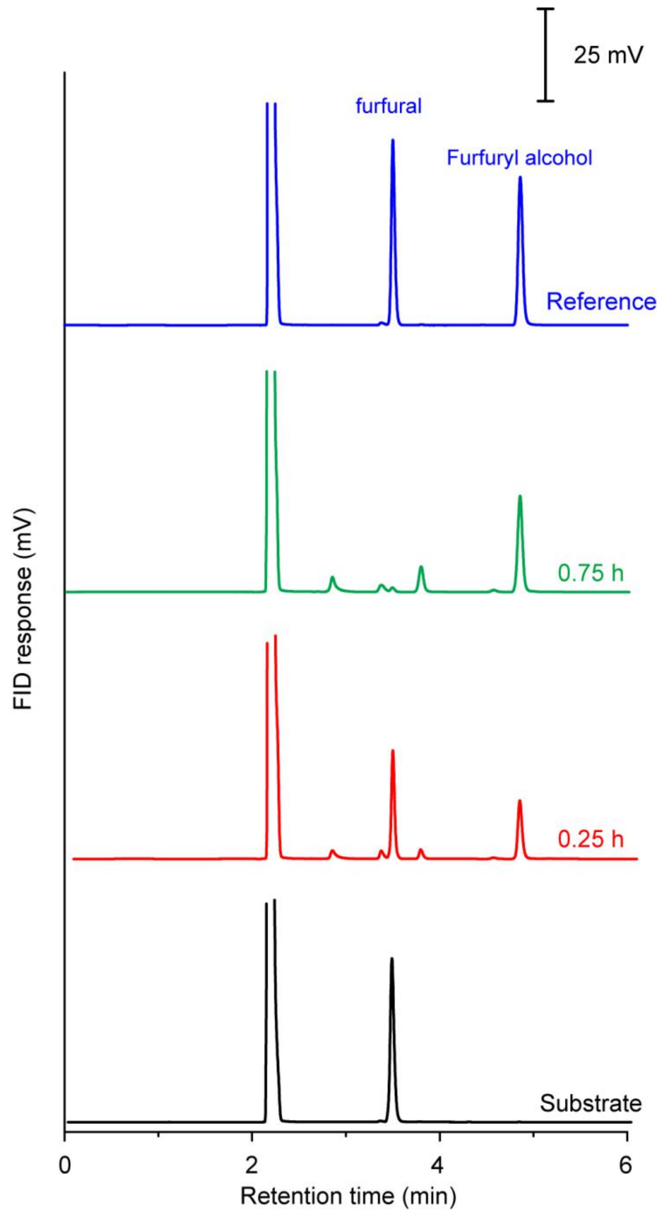

Supplementary Figure 18: The FID responded GC spectra of furfural substrate (black), the products obtained at 0.25 h (red) and 0.75 h (green) and reference (blue) with known substances. GC parameters of temperature: gasification chamber (240 ^{o}C), FID detector (260 ^{o}C) and column (160 ^{o}C).

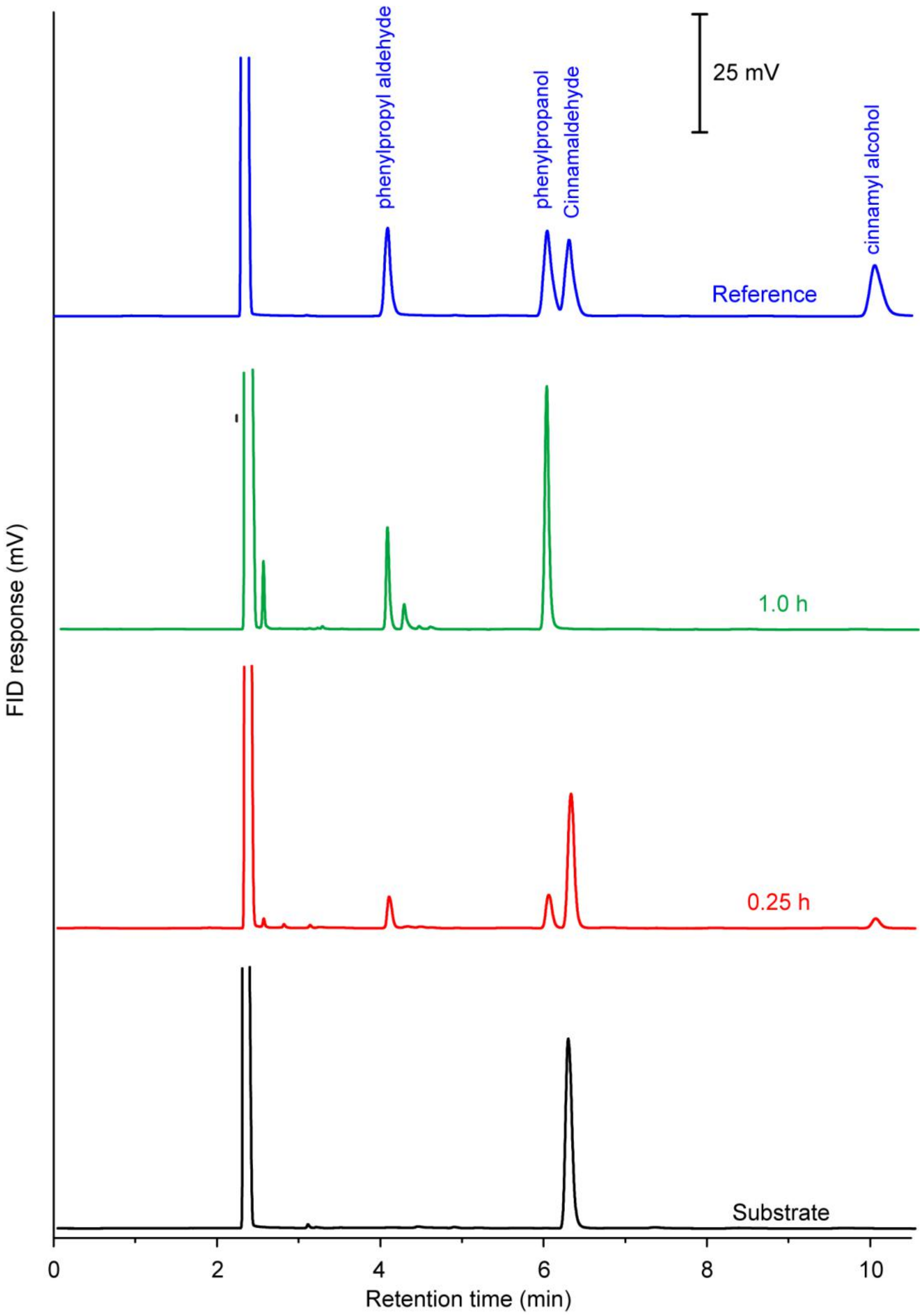

Supplementary Figure 19: The FID responded GC spectra of cinnamaldehyde substrate (black), the products obtained at 0.25 h (red) and 1 h (green) and reference (blue) with known substances. GC parameters of temperature: gasification chamber (240 ^{o}C), FID detector (260 ^{o}C) and column (200 ^{o}C).

.

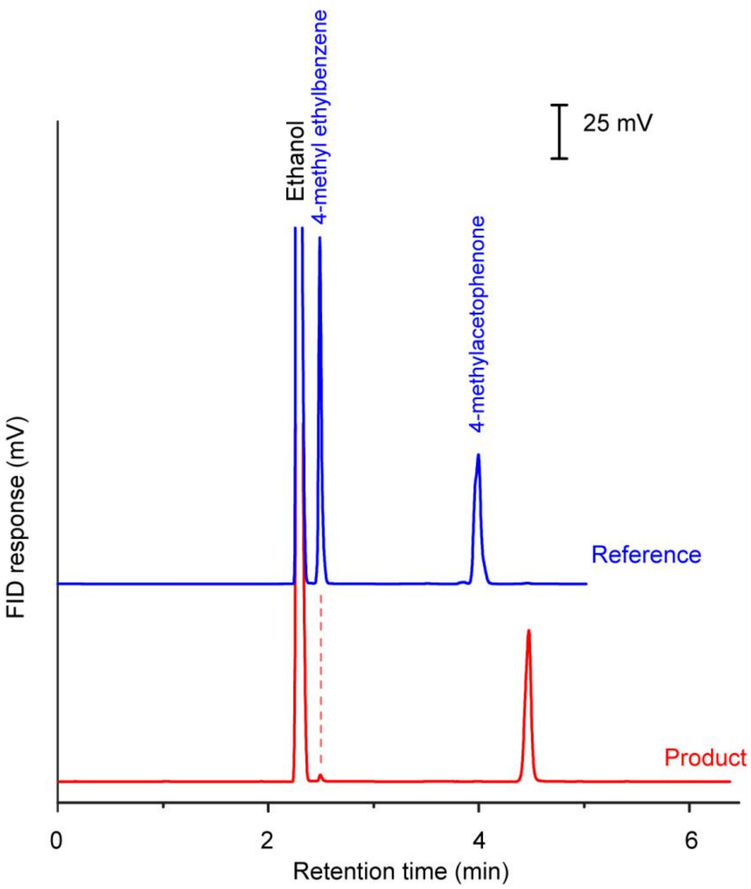

Supplementary Figure 20: The FID responded GC spectra of hydrogenation product (red) of 4-methylacetophene and reference (blue) with known substances. GC parameters of temperature: gasification chamber (240 ^{o}C), FID detector (260 ^{o}C) and column (200 ^{o}C).

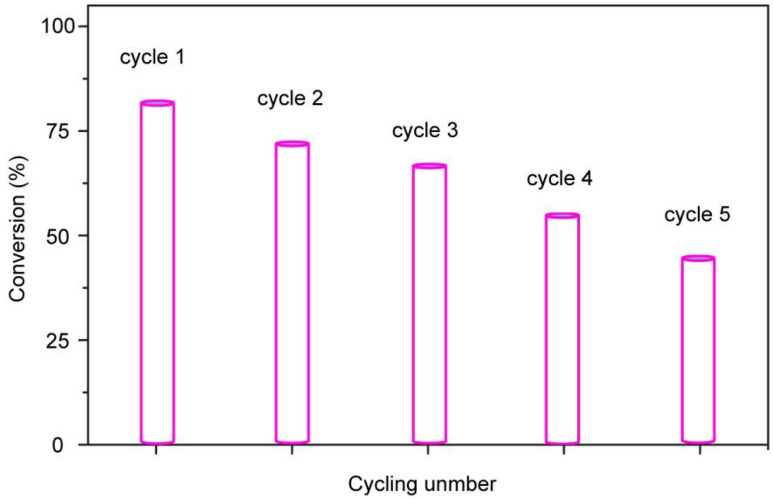

Supplementary Figure 21: The conversions of 4-methylacetophene at reaction time of 0.5 h in 5 cycles with 5% Pd_{1+NPs}/TiO_2 catalysts under 1 atm H_2 pressure at 25 ^oC. 5 mg of the catalysts were used. The H_2 flow was 10 mL/min.

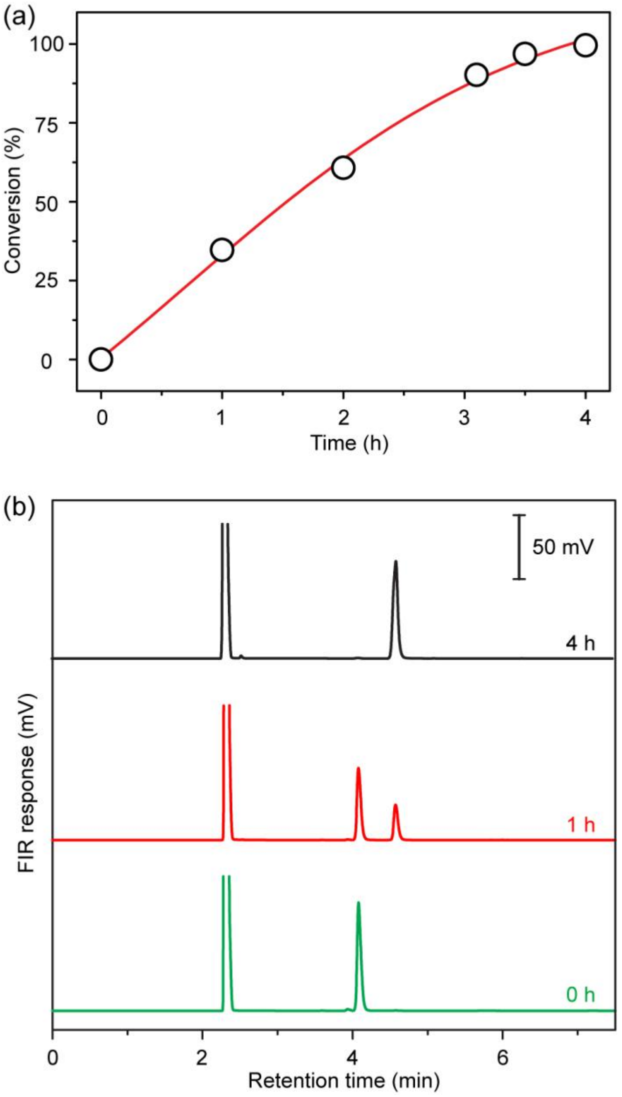

Supplementary Figure 22: 4-methylacetophene (MAP) conversion plots (a) and the FID responded GC spectra (b) with 5% Pd_{1+NPs}/TiO_2 catalysts under 1 atm H_2 pressure at 25 °C. 5 mg of the catalysts were used, and the substrates were increased to 5 mmol in 5 mL ethanol. The H_2 flow was 10 mL/min. GC parameters of temperature: gasification chamber (240 °C), FID detector (260 °C) and column (200 °C).

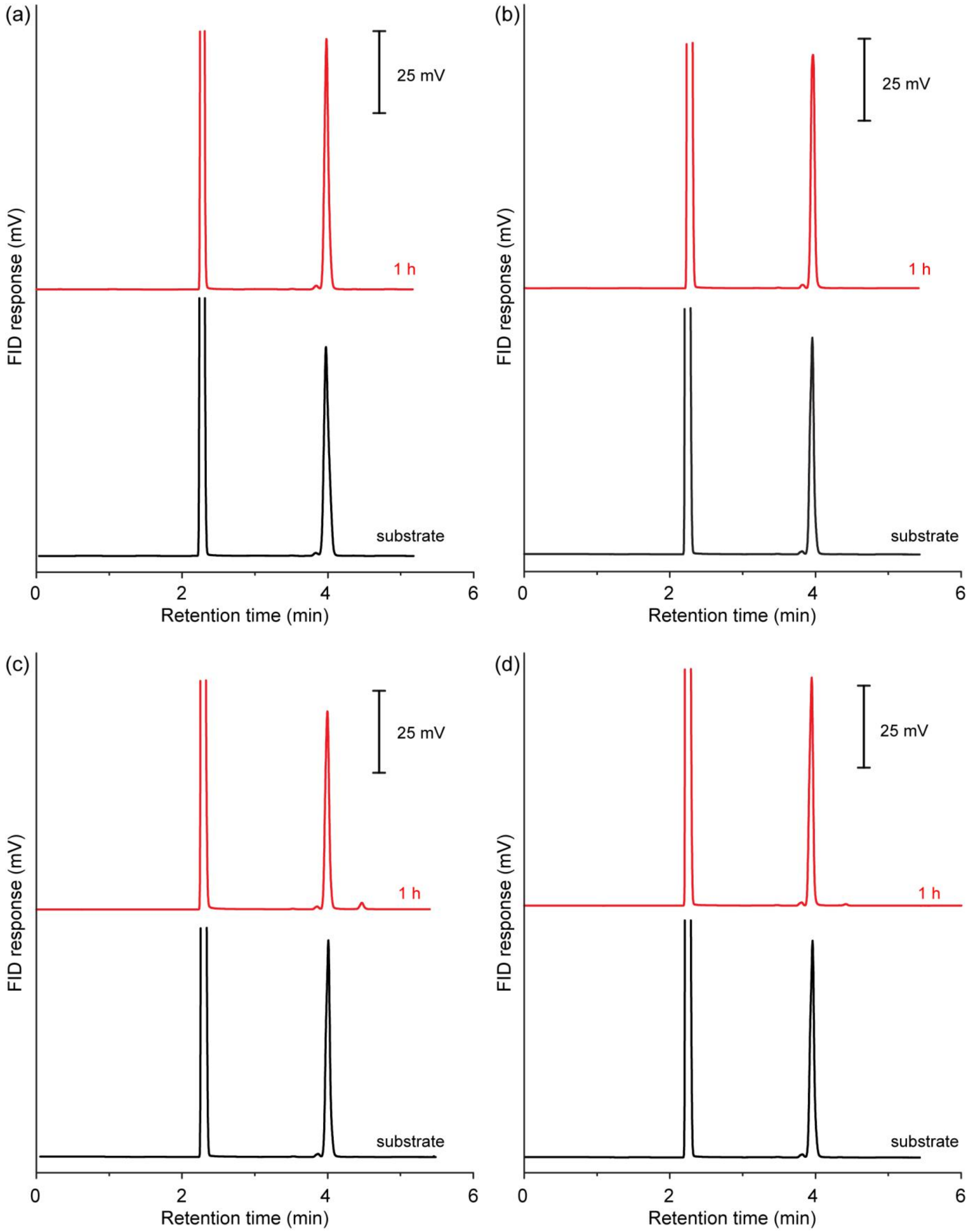

Supplementary Figure 23: The FID responded GC spectra of 4-methylacetophene substrate (black), the products obtained at 1 h (red) with 5% Pd/Al_2O_3 (a), Pd/Mn_3O_4 (b), Pd/NiO (c) and Pd/Fe_2O_3 (d) catalysts. GC parameters of temperature: gasification chamber (240 ^oC), FID detector (260 ^oC) and column (200 ^oC).

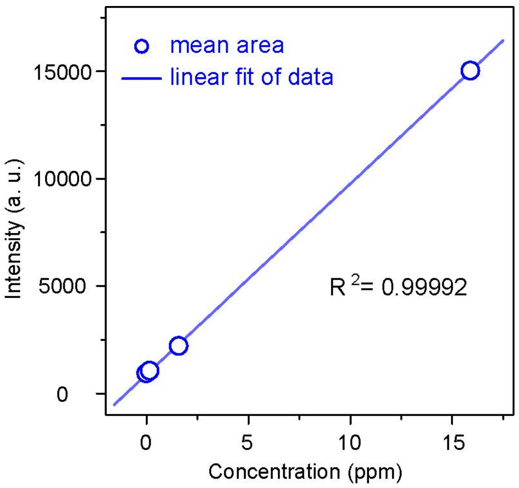

Supplementary Figure 24: The concentration-dependent intensity of Pd standard solutions from inductively coupled plasma-atomic emission spectrometry (ICP-AES).

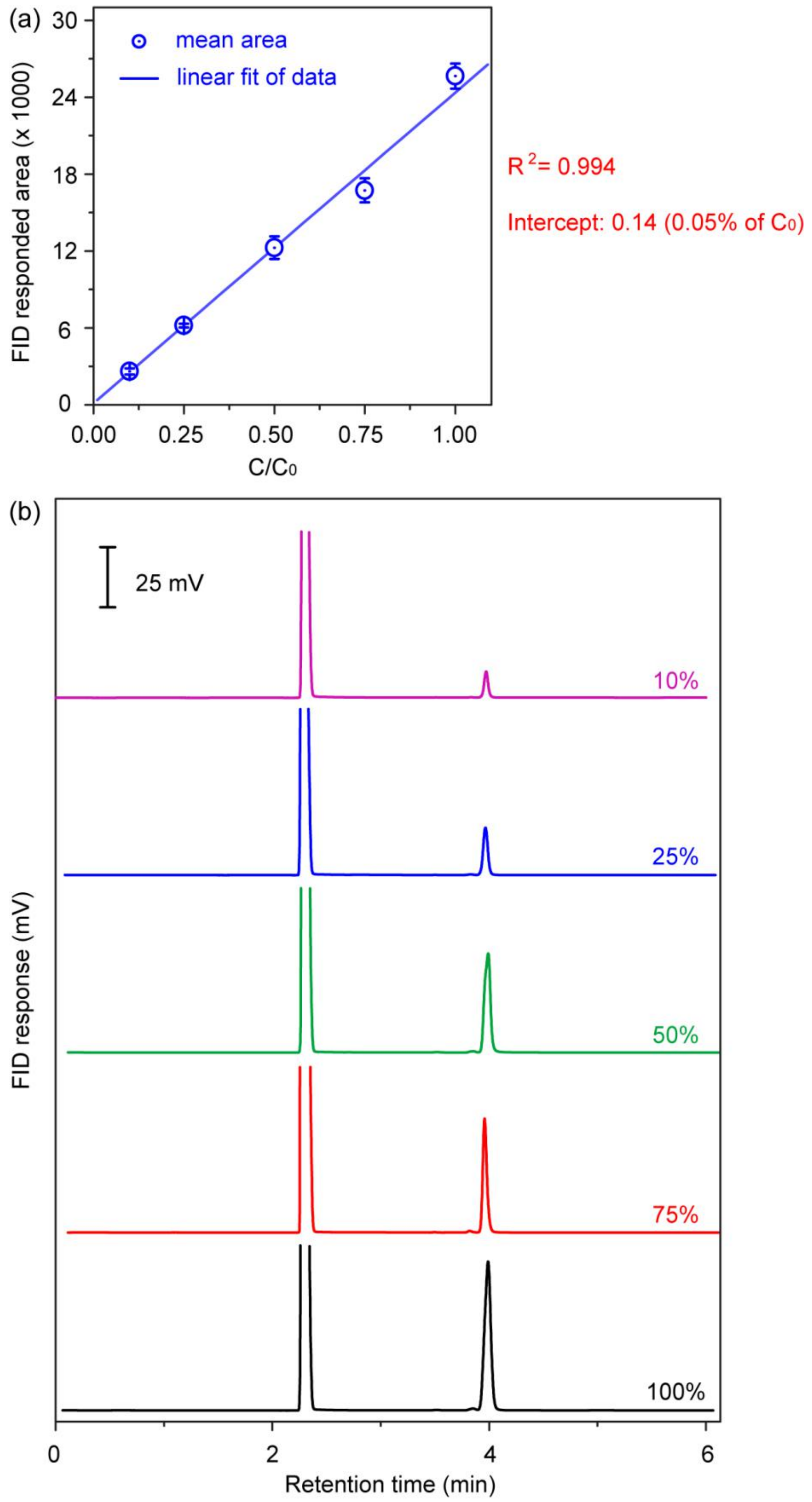

Supplementary Figure 25: (a) The concentration dependent FID responded and fitted areas of 4-methylacetophene; (b) The FID responded GC spectra of 4-methylacetophene with C/C_0 of 100% (black), 75% (red), 50% (green), 25% (blue) and 10% (purple). GC parameters of temperature: gasification chamber (240 ^oC), FID detector (260 ^oC) and column (200 ^oC). The C_0 of 4-methylacetophene is 0.2 mol/L (1 mmol 4-methylacetophene in 5 mL ethanol).

Supplementary Tables

Supplementary Table 1: Structural parameters of Pd_{1+NPs}/TiO_2, Pd_1/TiO_2 and Pd_{NPs}/TiO_2 catalysts extracted from the EXAFS fitting with references of Pd foil and bulk PdO. (S_0^2=0.80)

Sample	Scattering pair	CN	R(Å)	σ^2 (10^{-3}Å^2)	ΔE_0 (eV)	R factor
Pd_1/TiO_2	Pd-O	3.4±0.6	2.01±0.02	5.4±0.9	-4.3±1.2	0.0064
Pd_{1+NPs}/TiO_2	Pd-O	2.0±0.4	2.02±0.02	5.8±1.2	5.1±0.8	0.0075
	Pd-Pd	2.9±0.5	2.74±0.02	6.7±0.7	-3.2±0.7	0.0075
Pd_{NPs}/TiO_2	Pd-O	0.9±0.2	2.03±0.02	5.5±1.2	5.2±0.9	0.0083
	Pd-Pd	8.2±0.5	2.74±0.02	7.4±0.9	-7.2±1.3	0.0083
Pd foil	Pd-Pd	12	2.74±0.02	6.2±0.4	-4.2±0.7	0.0009
PdO	Pd-O	4	2.02±0.02	5.4±0.7	5.5±2.5	0.0061
	Pd-Pd	4	3.01±0.02	5.6±1.4	-3.5±0.4	0.0018
	Pd-Pd	8	3.41±0.02	5.3±1.1	-3.8±0.4	0.0018

S_0^2 is the amplitude reduction factor; CN is the coordination number; R is interatomic distance (the bond length between central atoms and surrounding coordination atoms); σ^2 is Debye-Waller factor (a measure of thermal and static disorder in absorber-scatter distances); ΔE_0 is edge-energy shift (the difference between the zero kinetic energy value of the sample and that of the theoretical model). R factor is used to value the goodness of the fitting.

Supplementary Table 2: The performance summary of various catalysts toward 4-methylacetophenone hydrogenation.

Item / Sample	Conversion[a] %	$S_{Alcohol}$[b] %	Dispersion %	TOF value h^{-1}
4.8% Pd_{1+NPs}/TiO_2	100	99	17.7	4362
Pd_{NPs}/TiO_2	50.0	>99	6.2	4565
2.8% Pd_{1+NPs}/TiO_2	64.0	99.8	22.0	1341
Pd_1/TiO_2	73.6	>99	67[c]	645
Commercial Pd/C	42.6	96[d]	32.8	1218
TiO_2	N. D.[e]	N. D.	/	/
Catalyst-free	N. D.	N. D.	/	/

[a]The catalytic evaluation was performed at 1 hours' reaction time with 1 mmol substrate in 5 mL ethanol under 1 atm H_2 pressure at 25 ^{o}C. The ratio of Pd to 4-methylacetophenone substrate is 0.2 mol%.

[b]$S_{Alcohol}$ means the alcohol selectivity.

[c]The Pd dispersion were estimated according to Pratsinis et al.' work[1] as 67% by leaching surface Pd with mixture of H_2O_2/HCl solution.

[d]The alcohol selectivity would further drop when the conversion reaches 100%.

[e]'N. D.' means 'Not detected'.

Supplementary Table 3: The adsorption energies of C_8H_8O and H_2 molecules on the surface of Pd (111) and Pd_1/TiO_2(110) based on DFT calculation.

System	ΔE (C_8H_8O)/eV	ΔE (H_2)/eV
Pd(111)	-0.34194	-0.08831
Pd_1/TiO_2(110)	-0.91719	-0.15283

Supplementary notes

Note to Supplementary Fig. 1. The as-prepared Pd_{1+NPs}/TiO_2 synergistic catalyst presents mesoporous microspheres (Supplementary Fig. 1a and b). Several nanosized Pd particles (darker points) embedded in the TiO_2 matrix can be found in the TEM image. The atomically dispersed Pd sites cannot be identified in the TEM image, but it can be confirmed by AC-HAADF-STEM image and EXAFS spectra, which can be seen in Figure 1b and c in the main text. According to the XRD pattern (Supplementary Fig. 1c), the TiO_2 support presents anatase phase (JCPDF card NO. 1-562) and the Pd NPs (highlighted by pink circles) appears face-centered cubic phase (JCPDF card NO. 1-1202).

Note to Supplementary Fig. 3. As shown in Figure S3a, Based on Supplementary Fig. 3a and b, Pd nanoparticle (~ 10 nm) was clearly found, which were highlighted by yellow square in Supplementary Fig. 3b. The atomically scaled images (Supplementary Fig. 3c-e) clearly presented the Pd single-atoms (the brighter spots highlighted by red circles.). Meanwhile, a Pd raft was also found.

Note to Supplementary Fig. 7. Based on Supplementary Fig. 7a and b, no Pd nanoparticle was found. The atomically scaled images (Supplementary Fig. 7c-e) clearly presented the Pd single-atoms (the brighter spots highlighted by red circles.).

Note to Supplementary Fig. 8. According to Supplementary Fig. 8a-c, Pd nanoparticles (brighter spots in Figure S8a and b, brighter particles in Figure S8c) were clearly presented for 5% Pd_{NPs}/TiO_2 mesoporous catalysts. No Pd cluster can be found in the lower magnification of atomically scaled image (Supplementary Fig. 8d). Furthermore, the surface of TiO_2 support was clean and no Pd single atom can be found.

Note to Supplementary Fig. 11. Based on Supplementary Fig. 11a and b, no Pd nanoparticle was found in used 1% Pd_1/TiO_2 catalysts. The atomically scaled images (Supplementary Fig. 11c) clearly presented the Pd single-atoms (the brighter spots highlighted by red circles). In Supplementary Fig. 11d and e, we also found that a few Pd_1 atoms aggregated to subnano cluster (highlighted by yellow circles).

Note to Supplementary Fig. 13. The blue curve in Supplementary Fig. 13 is the reference spectrum with ethylbenzene, acetophenone and α-phenethyl alcohol with mole ratio of 1.058 : 1.427 : 1 and corresponding peak area ratio of 1.319 : 1.422 : 1.

Note to Supplementary Fig. 18. The blue curve in Supplementary Fig. 18 is the reference spectrum with furfural and furfuryl alcohol with mole ratio of 1 : 1.053 and corresponding peak area ratio of 1 : 1.052.

Note to Supplementary Fig. 19. The blue curve in Supplementary Fig. 19 is the reference spectrum with phenylpropyl aldehyde, phenylpropanol, cinnamaldehyde and cinnamyl alcohol with mole ratio of 1 : 1 : 1 : 1 and corresponding peak area ratio of 1 : 1.221: 1.133 : 1.060.

Note to Supplementary Fig. 20. The blue curve in Supplementary Fig. 20 is the reference spectrum with 4-methyl ethylbenzene and 4-methylacetophene with mole ratio of 1.023 : 1 in 5 mL ethanol. Compared with the reference GC spectra, it is concluded that the substance with retention time of 2.55 min is assigned to the 4-methyl ethylbenzene, which is produced by hydrodeoxygenation of 4-methylacetophene. In addition, the selectivity of 4-methyl ethylbenzene is herein < 1% based on the peak area.

Note to Supplementary Fig. 21. To evaluate stability of Pd_{1+NPs}/TiO_2 catalysts, the conversion at 0.5 h at each cycle was considered. Because the catalyst amount (5 mg) was quite low, we didn’t separate the catalysts from the reaction system. To decrease the catalyst loss, pure substrate was added into the reaction cell after completing the last reaction (1 h). As shown in Supplementary Fig. 21, the reactivity drops gradually with the increase of cycle. At 5th cycle, 45% of the reactivity was lost in comparison with the first cycle. It was worth to mention that the reactivity of catalysts was also affected by the chemical equilibrium of the accumulated product because the product was also not separated out.

Supplementary References

1. Fujiwara, K.; Pratsinis, S. E. Single Pd atoms on TiO_2 dominate photocatalytic NO_x removal. *Appl. Catal. B: Environ. 226*, 127-134 (2018).
